# Type IV collagen is a tumour stroma-derived biomarker for pancreas cancer

**DOI:** 10.1038/sj.bjc.6605107

**Published:** 2009-06-02

**Authors:** D Öhlund, C Lundin, B Ardnor, M Öman, P Naredi, M Sund

**Affiliations:** 1 Department of Surgical and Perioperative Sciences, Surgery, Umeå University, Umeå SE-901 85, Sweden

**Keywords:** extracellular matrix, basement membrane, surgery, circulation, biomarker, pancreas

## Abstract

**Background:**

Pancreas cancer is a dreaded disease with high mortality, despite progress in surgical and oncological treatments in recent years. The field is hampered by a lack of good prognostic and predictive tumour biomarkers to be used during follow-up of patients.

**Methods:**

The circulating level of type IV collagen was measured by ELISA in pancreas cancer patients and controls. The expression pattern of type IV collagen in normal pancreas, pancreas cancer tissue and in pancreas cancer cell lines was studied by immunofluorescence and Western blot techniques.

**Results:**

Patients with pancreas cancer have significantly increased circulating levels of type IV collagen. In pancreas cancer tissue high levels of type IV collagen expression was found in close proximity to cancer cells in the tumour stroma. Furthermore, pancreas cancer cells were found to produce and secrete type IV collagen *in vitro*, which in part can explain the high type IV collagen expression observed in pancreas cancer tissue, and the increased circulating levels in pancreas cancer patients. Of clinical importance, our results show that the circulating level of type IV collagen after surgery is strongly related to prognosis in patients treated for pancreas cancer by pancreatico-duodenectomy with curative intent. Persisting high levels of circulating type IV collagen after surgery indicates a quick relapse in disease and poor survival.

**Conclusion:**

Our results most importantly show that stroma related substances can be evaluated as potential cancer biomarkers, and thereby underline the importance of the tumour microenvironment also in this context.

Pancreas cancer is still one of the most feared malignancies with a poor prognosis. The overall median survival is 3–5 months, and the 5-year survival rate is only 3% (Goldstein *et al*, 2004). This is due to unspecific and diffuse symptoms, leading to late diagnosis of the disease. Currently, the only known curative treatment is surgical removal of a tumour at a local stage, and the procedure most often used to treat cancerous tumours in the head of the pancreas is pancreatico–duodenectomy (Whipple's procedure). In Whipple's procedure the distal half of the stomach (optional), the gall bladder, the distal portion of the common bile duct, the head of the pancreas, duodenum, proximal jejunum and regional lymph nodes are all removed. Although the results in surgical treatment of pancreatic cancer have improved over the last decades, long-term survival occurs in only 10–20% of patients (Goldstein *et al*, 2004). In fact, many patients diagnosed with a local operable tumour turn out to suffer from spread disease. Taken together, this indicates that there is a great need for new biomarkers to assist in obtaining an early and accurate diagnosis, classify patients correctly as having local (operable) or spread (non-operable) disease. A good biomarker would also aid in evaluating treatment effects, predicting prognosis and monitoring patients after treatment for early detection of disease relapse.

Several different substances have been identified as potential biomarkers in pancreas cancer, such as LMO4 (Murphy *et al*, 2008), c-kit (Kimura *et al*, 2007), TPS, CA 19-9, VEGF-A and CEA (Slesak *et al*, 2004; Sandblom *et al*, 2008) in serum, and telomerase activity in pancreatic juice (Uehara *et al*, 2008). However, few of these are used in clinical praxis, and the specificity and sensitivity for many have been questioned.

A characteristic feature of pancreas cancer is the dens fibrous stroma surrounding the cancer cells and referred to as tumour desmoplasia. It has been suggested that desmoplasia is of importance for cancer cell growth and survival, which is supported by the fact that the less aggressive mucinous type of pancreatic carcinoma is not surrounded by such an abundant desmoplastic stroma (Kimura *et al*, 1998). This indicates that the extracellular matrix (ECM) is of particular interest in pancreas cancer.

Type IV collagen is an ECM protein found in all basement membranes (BM) (Khoshnoodi *et al*, 2008). Type IV collagen forms a supramolecular network in the basement membrane that influences cell adhesion, migration and differentiation of epithelial cells (Khoshnoodi *et al*, 2008). This BM protein can be made from six different *α*-chains, *α*1(IV)–*α*6(IV), which appear in different combinations to form triple helical molecules called protomers (Hudson *et al*, 2003) that further organise into a sheet-like structure in the BM. Tissues show unique expression patterns of *α*(IV)-chains, which renders different functional and structural properties to the BMs in that tissue.

In recent years it has been shown that fragments from type IV collagen can act as endogenous antiangiogenic agents. Arresten, canstatin and tumstatin (fragments derived from *α*1(IV)-, *α*2(IV)- and *α*3(IV)-chains, respectively) have been shown to inhibit angiogenesis and tumour growth *in vivo* (Kamphaus *et al*, 2000; Kalluri, 2003; Sund *et al*, 2005). In addition, fragments derived from *α*4(IV)-, *α*5(IV)- and *α*6(IV)-chains were found to have antiangiogenic features *in vitro* (Karagiannis and Popel, 2007). Although the role of type IV collagen in angiogenesis and cancer progression has been extensively studied, few have evaluated this protein as a potential biomarker. However, a correlation between levels of type IV collagen in peritoneal fluid and survival time in patients with peritoneal disseminated gastric and colorectal cancer has been shown (Korenaga *et al*, 1998).

We show here the expression pattern of different *α*(IV)-chains in normal pancreas and pancreatic cancer tissue, as well as analyse the expression of type IV collagen by pancreas cancer cells *in vitro*. We also measure the circulating levels of type IV collagen in patients with pancreas cancer before and after treatment, and introduce type IV collagen as a promising future biomarker for identification of patients at high risk of quick relapse and poor prognosis after surgery.

## MATERIALS AND METHODS

### Patients and samples

Patients with pancreas cancer were admitted to the Department of Surgery, Umeå University Hospital and evaluated by CT scan, MRI and laparoscopy. Patients who were considered operable underwent a curatively aimed pancreatico–duodenectomy (Whipple's procedure). One patient was operated with an expanded Whipple's procedure (total pancreatectomy), and one obtained intraoperative radiotherapy (IORT). Plasma samples were collected from these patients before (Pre Op; *n*=9) and after surgery (Post Op, *n*=9). In the survival analysis additional patients were added (*n*=5), where only samples after surgery were available. Postoperative plasma samples were collected 4 weeks or more after surgery. Control samples were collected from patients (*n*=8) admitted to the hospital for a non-malignant disease. Patient characteristics are shown in Table 1. The samples were frozen and stored at −80°C until analysis. Pancreas tissue samples from patients with pancreas cancer (*n*=6) and controls (*n*=5) were snap frozen in liquid nitrogen and stored at −80°C until analysis. Informed consent was obtained from all patients. The ethical committee at the Medical faculty of Umeå University approved the study.

### Cell culture

Two well characterised human pancreatic adenocarcinoma cell lines were used, HPAC and CFPAC-1. Both cells lines were kindly provided by Professor Helena Edlund, Umeå University. HPAC (ATCC, CRL-2119) is a human pancreatic adenocarcinoma cell line of ductal origin from a woman with moderate to well differentiated pancreatic adenocarcinoma (Gower, 1994). CFPAC-1 (ATCC, CRL-1918) is derived from a ductal adenocarcinoma from a male patient with cystic fibrosis (Schoumacher *et al*, 1990). HPAC were cultured with 1 : 1 mixture of Dulbecco's modified Eagle's and Ham's F12 medium (DMEM/F12), CFPAC-1 with 90% Iscove's modified Dulbecco's medium (IMDM), both supplemented with 10% fetal bovine serum, penicillin (10^5^ IU l^−1^) and streptomycin (100 mg l^−1^) and grown in an incubator at 37°C in an atmosphere of 5% CO_2_.

### Tissue and cell staining

Hematoxylin and eosin (H&E) and Masson's trichrome staining (MTS) were performed according to standard protocols. Immunohistochemical staining was performed on frozen sections (six micrometer) and immunocytochemical staining on HPAC and CFPAC-1 cells cultured on Falcon Culture Slides (BD Biosciences, Erembodegem, Belgium) using methods described earlier (Hamano *et al*, 2003) with the following primary antibodies and dilutions; mouse-anti-*α*1(IV)NC1 (Wieslab, Malmo, Sweden, MAB1, 1 : 75) (Johansson *et al*, 1991), mouse-anti-*α*2(IV)7S (Chemicon, Billerica, MA, USA, MAB1910, 1 : 400), mouse-anti-*α*3(IV)NC1 (Wieslab MAB3, 1 : 75) (Johansson *et al*, 1991), mouse-anti-*α*5(IV)NC1 (Wieslab MAB5, 1 : 75) (Ding *et al*, 1994), rabbit-anti-collagen type IV (polyclonal, MP Biomedicals LLC, Illkirch, France) and sheep-anti-CD31 (R&D systems, Abingdon, UK). Unfortunately, no specific antibodies against the *α*4(IV)- and *α*6(IV)-chains are commercially available. For detection of *α*5(IV)NC1, tissue was denatured with 6 M urea–0.1 M glycine before staining. Secondary antibodies used were; donkey-anti-sheep FITC (Jackson ImmunoResearch lab Inc., Newmarket, UK, 1 : 100), and donkey-anti-mouse TRITC (Jackson ImmunoResearch lab Inc., 1:100) and donkey-anti-rabbit FITC (Jackson ImmunoResearch lab Inc., 1 : 100). Sections were mounted with medium containing DAPI (Vectashield, Vector Laboratories Inc.). Positive control staining for each antibody was performed on tissue known to express the antigen in question. As negative controls, primary antibodies were omitted and sections were incubated with secondary antibodies only.

### Western blot

HPAC and CFPAC cells were cultured to subconfluency, then serum-starved for 1–2 days. Cells were lysed as described earlier (Aebi *et al*, 1996). The proteins secreted to the cell media were collected by ethanol precipitation and dissolved in reducing buffer. In all, 20 *μ*g of protein from the cell layer and 25 *μ*g of protein from the cell media were loaded onto a 12% SDS–polyacrylamide gel. As a positive control 500 ng of recombinant human arresten (for *α*1(IV)NC1) (Colorado *et al*, 2000) and 100 ng canstatin (for *α*2(IV)NC1) (Kamphaus *et al*, 2000) were used. Recombinant protein was kindly provided by Professor Raghu Kalluri, HMS, Boston. A rabbit-anti-type IV collagen (1 : 500) (Kamphaus *et al*, 2000) antibody, detecting both *α*1(IV)- and *α*2(IV)-chains, was used as a primary antibody, and peroxidase-conjugated goat-anti-rabbit-IgG (Sigma, Stockholm, Sweden, A 9169, 1:12000) as secondary antibody. Proteins were detected by chemiluminescense (ECL) (GE Healthcare, Uppsala, Sweden).

### ELISA assay

Circulating levels of type IV collagen in patients and controls were measured by using Serum Collagen IV EIA (Argutus Medical, Dublin, Ireland). Samples were run in duplicates and according to the manufacturer's protocol. In these ELISA-assays two monoclonal antibodies (clones 4H12 and 1D3) directed against the collagenous domain of type IV collagen are used (Obata *et al*, 1989).

### Statistics

Statistical analysis when comparing different groups were performed by using the Student's *t*-test. The variance of groups was tested for equality by an *F*-test prior to a *t*-test analysis. A two-tailed analysis was used throughout. Values in text and figures are presented as mean±s.d. SPSS version 16.0 was used for regression and survival analysis.

## RESULTS

### High expression of type IV collagen is observed in the tumour stroma

**Figure 1:**
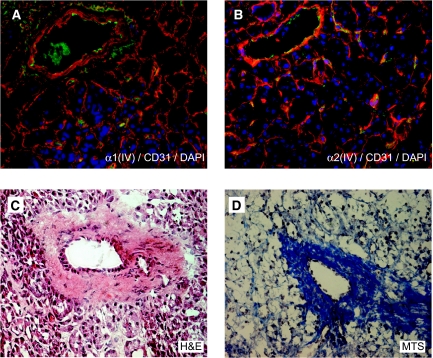
Expression of type IV collagen in normal pancreas. (**A**, **B**) In normal pancreas tissue *α*1(IV)- and *α*2(IV)-chains are expressed in epithelial and vascular BMs (in red). Blood vessels are visualised by the endothelial cell marker CD31 (in green) and nuclei by DAPI (in blue). (**C**, **D**) Light microscopy images stained with H&E (**C**) and MTS (**D**) of the corresponding areas in normal pancreas. In (**D**) MTS collagen fibres are stained blue. Magnification × 40 in all panels.

**Figure 2:**
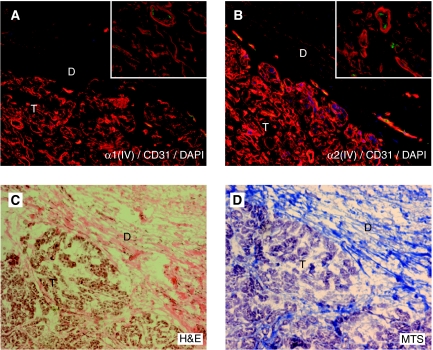
Expression of type IV collagen in pancreas cancer. (**A**, **B**) In pancreas cancer tissue *α*1(IV)- and *α*2(IV)-chains are strongly expressed (in red) in the tumour stroma (marked T) and in the VBM, whereas the expression is low in the surrounding desmoplastic area that contains an abundance of fibrillar collagens (marked D). Blood vessels are visualised by the endothelial cell marker CD31 (in green) and cell nuclei by DAPI (in blue). Insets show a close-up of the tumour stroma. (**C**, **D**) Light microscopy images stained with H&E (**C**) and MTS (**D**) of the corresponding areas in pancreas cancer. In (**D**) MTS the collagen fibres are stained blue. Magnification × 20 in all panels and × 40 in inserts.

We analysed the expression pattern of the different *α*-chains of type IV collagen in normal pancreas and in pancreas cancer tissue. In normal pancreas *α*1(IV)- and *α*2(IV)-chains are highly expressed in vascular basement membranes (VBM) and epithelial basement membranes (Figure 1), whereas the *α*3(IV)- and *α*5(IV)-chains could not be detected (data not shown). In pancreas cancer the tissue morphology is changed, with no organised ductal structures, and strong *α*1(IV)- and *α*2(IV)-chain expression is now observed close to the cancer cells in the tumour stroma and the VBMs (Figure 2). Interestingly, in the desmoplastic reaction surrounding the tumour, only low levels of type IV collagen expression could be observed. The *α*3(IV)- and *α*5(IV)-chains of type IV collagen were not expressed in pancreas cancer (data not shown).

### Type IV collagen is produced by pancreas cancer cells *in vitro*

**Figure 3:**
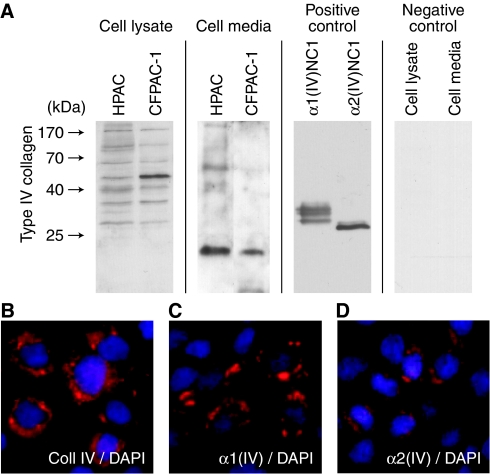
Expression of type IV collagen in pancreas cancer cell lines. (**A**) Cell lysates of HPAC and CFPAC-1 contain type IV collagen-derived fragments, demonstrating production of this collagen by cells. The complete *α*(IV)-chain is too large to enter the gel, and the bands visualised correspond to fragments of different sizes cleaved off from the *α*-chain in a constantly ongoing turnover process involving different tumour-associated proteases. In the cell media type IV collagen-derived fragments could also be observed in both cell lines, indicating secretion of type IV collagen from the cells to the media. As positive controls recombinant human arresten (*α*1(IV)NC1) and canstatin (*α*2(IV)NC1) were used. As negative control HPAC cell lysate and cell media were incubated without primary antibody. (**B**–**D**) Immunocytochemical analysis of HPAC and CFPAC-1 cells confirm that type IV collagen is produced by pancreas cancer cell lines. In (**B**) type IV collagen is visualised using a polyclonal antibody, in (**C**) an antibody specific for the *α*1(IV)-chain is used and in (**D**) an antibody specific for the *α*2(IV)-chain. Type IV collagen is shown in red and cell nuclei in blue (DAPI). Magnification × 40 in **B**–**D**.

To further study whether the observed type IV collagen is solely expressed by the stromal cells, such as the stellate cells surrounding the cancer cells in the tumour, or partly by the cancer cells themselves, we performed *in vitro* studies on two well characterised pancreas cancer cell lines HPAC and CFPAC-1. Western blot analysis of cell lysate and media, and immunocytochemical staining, show that both *α*1(IV) and *α*2(IV), the two *α*-chains that were strongly expressed in the tumour stroma in pancreas cancer tissue, are produced by the cancer cells and secreted to the media in both cell lines (Figure 3). These results suggest that the type IV collagen observed in the tumour stroma *in vivo* could be, at least in part, produced by the pancreas cells themselves.

### Changes in circulating levels of type IV collagen in patients with pancreas cancer

**Figure 4:**
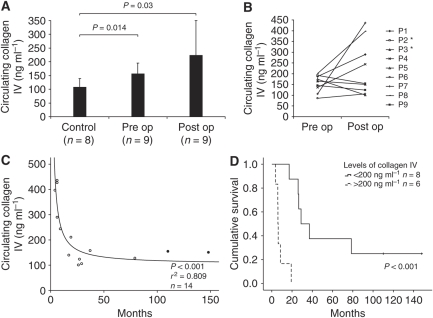
Change in circulating levels of type IV collagen in patients with pancreas cancer and correlation to survival. (**A**) In pancreas cancer patients circulating type IV collagen levels are increased compared with controls with non-malignant disease (*P*=0.014). After surgery the circulating levels remain high (*P*=0.03 *vs* controls), but error bars indicate great variance within the postoperative group. Values are presented as mean±s.d. (**B**) Pre- and postoperative levels of circulating type IV collagen in individual patients. Five patients show increased levels after surgery, four decreased. Asterisk (^*^) indicates papillary carcinomas. (**C**) Postoperative levels of type IV collagen correlate to survival (*P*<0.001, *r*^2^=0,809, *n*=14). High postoperative levels correlate to short survival. Closed circle (•) indicates patients that are alive (papillary carcinomas). (**D**) Kaplan–Meier survival curve. Patients with high levels of circulating type IV collagen after surgery (cutoff=200 ng ml^−1^) show significantly shorter survival than patients with low levels, *P*<0.001 (log-rank test). The cross (+) indicates patients still alive (papillary carcinomas).

Circulating levels of type IV collagen fragments were measured with ELISA in control patients and pancreas cancer patients before and after surgery (Figure 4A). A significant increase of circulating type IV collagen could be observed preoperatively when compared with controls 106±32 *vs* 154±40 ng ml^−1^. After surgery the type IV collagen levels remained high (222±127 ng ml^−1^) when compared with the controls, and no statistically significant change was observed when comparing with the levels before surgery. However, the variation within the postoperative group was high, which prompted us to plot the levels before and after surgery for individual patients (Figure 4B). It turned out that for five patients the levels increased after surgery and for four the levels decreased.

### Postoperative levels of type IV collagen correlates to survival

This observation made it interesting to look further into whether the observed differences in circulating type IV collagen levels after surgery are reflected in differences in prognosis and survival. We therefore plotted postoperative type IV collagen levels against registered survival after surgery (Figure 4C). At this point, five patients for whom only samples after surgery are available were added to the postoperative group (Table 1). We observed a significant correlation, where high circulating levels of type IV collagen after surgery corresponded to poor prognosis and short survival time. The difference in survival between patients with high postoperative levels of type IV collagen (>200 ng ml^−1^), and those with lower levels (<200 ng ml^−1^) can also be illustrated in a survival curve (Figure 4D). Median survival time in the group with persistent high levels of circulating type IV collagen is 6.1 months after surgery, compared with 28.9 months in the group with low levels. No correlation between total bilirubin levels, C-reactive protein levels and type IV collagen levels were observed (data not shown). Preoperative levels of circulating type IV collagen were also tested for correlation to survival, but no significant correlation could be observed in this case (data not shown).

## DISCUSSION

It has previously been shown that in the BM of the pancreatic ducts *α*1(IV)-, *α*2(IV)-, *α*5(IV)- and *α*6(IV)-chains are expressed in normal pancreas, whereas the *α*5(IV)- and *α*6(IV)-chains are lost from the BM in the pancreatic duct in cancer (Kadono *et al*, 2004). In this study, we analysed the expression pattern of type IV collagen in the exocrine part and distal pancreatic ducts of the normal pancreas and in pancreas cancer. In the normal pancreas we could observe both *α*1(IV)- and *α*2(IV)-chain staining in the BM surrounding the acini structures and distal ducts in the exocrine pancreas. This suggests the presence of the *α*1*α*2*α*2 protomer of type IV collagen in this part of the normal pancreatic tissue, as we could not see any expression of the *α*3(IV)- and *α*5(IV)-chains.

In pancreas cancer, high levels of *α*1(IV)- and *α*2(IV)-chains were expressed in the direct proximity to the cancer cells in the tumour stroma. No expression of the *α*3(IV)- and *α*5(IV)-chains were observed. This is well in line with earlier studies of the expression pattern of type IV collagen in pancreatic adenocarcinomas (Lee *et al*, 1994; Linder *et al*, 2001). The typical strong desmoplastic reaction that surrounds the tumour cells in pancreas cancer is composed of connective tissue, predominantly type I collagen (Goldstein *et al*, 2004). Most interestingly, only low levels of *α*1(IV)- and *α*2(IV)-chain expression was found in the areas of tumour desmoplasia, indicating that type IV collagen has a distinct expression pattern when compared with the fibrillar collagens found in this area.

As an adenocarcinoma develops and becomes invasive it has to penetrate the BM. In this process matrix metalloproteinases (MMPs) are involved in remodelling the basement membrane (Stallings-Mann and Radisky, 2007). We have previously shown an upregulated expression of MMP-3 and MMP-9 in pancreas cancer tissue (Ohlund *et al*, 2008), and interestingly both these proteases have type IV collagen as a substrate (Nyberg, 2005). The altered expression pattern of type IV collagen observed in pancreas cancer tissue can therefore partly be due to disruption and reorganisation of pre-existing BMs in the tumour area by MMPs, but this would not wholly explain the expression pattern observed. The expression pattern of type XVIII collagen/endostatin, another component of the BM, is also altered in pancreas cancer (Ohlund *et al*, 2008). However, the expression pattern of type XVIII collagen does not resemble the expression pattern for type IV collagen in pancreas cancer. For type XVIII collagen the expression in tumour stroma is lower than that for type IV collagen, which indicates that various components of the ECM could have specific roles in the regulation of the tumour microenvironment of a cancer. Our findings also led us to the conclusion that a part of the observed type IV collagen must be produced by either stromal cells and/or cancer cells. In our subsequent *in vitro* experiments on two pancreas cancer cell lines, we could verify that pancreas adenocarcinoma cells produce and secrete type IV collagen. Our results therefore indicate that the expression pattern of type IV collagen observed in pancreas cancer tissue is a result of remodelling of pre-existing type IV collagen, together with *de novo* production by the cancer cells. It has been shown *in vitro* in other human cancer cells, such as glioblastoma and melanoma cell lines, that type IV collagen is produced by the cancer cell (Bouterfa *et al*, 1999). Most interestingly, it has been reported that type IV collagen production exceeds that of other well known ECM proteins, in several different pancreas cancer cell lines (Lohr *et al*, 1994). This further underlines the possible importance of type IV collagen in progression of pancreas cancer.

This is the first description of increased circulating type IV collagen levels in patients with pancreas cancer. We believe that this increase is due to the breakdown of existing BMs by the tumour, combined with type IV collagen production and secretion by the cancer cells. Furthermore, we show that circulating type IV collagen levels have prognostic value when predicting prognosis after surgery with curative intent. On the other hand, preoperative circulating type IV collagen levels do not predict prognosis. This is most likely due to the fact that preoperative level of circulating type IV collagen corresponds to the total tumour load. One can speculate that a large primary tumour can produce high levels of type IV collagen, because of both matrix remodeling and expression by the cancer cells, but it can nevertheless be a locally growing tumour with good prognosis. After successful removal of the primary tumour, the type IV collagen levels instead reflect the metastatic tumour load, and high levels in the postoperative situation therefore could correspond to spread disease and poor prognosis. Our findings indicate that by measuring circulating levels of type IV collagen soon after surgery, patients at high risk of quick relapse and poor prognosis can be identified in an early stage and be offered more aggressive adjuvant therapy.

Attachment to the BM is crucial for the survival and growth of epithelial cells. Integrin receptors play a role in anchoring the cells to the ECM and in providing survival signalling. If the epithelial cell looses contact with the BM, a cascade of events is initiated eventually leading to apoptosis (Hynes, 2002; Huveneers *et al*, 2007). In the transformation process from a local and benign, to an invasive and malignant cancer cell, this mechanism must be overrun, otherwise apoptosis will be induced as soon as the BM is broken down to make tumour growth possible. It was shown earlier that the integrin–ECM axis plays an important role in regulating growth and migration of pancreas cancer cells, and that many integrin receptors are upregulated in pancreas cancer (Grzesiak *et al*, 2007). Recent evidence also points at *α*_1_*β*_1_ integrin functioning as a type IV collagen receptor in pancreas cancer cells (Grzesiak and Bouvet, 2007). In this study, we show that high levels of type IV collagen is present in the close proximity to the cancer cells, and that cancer cells produce and secrete type IV collagen. We hypothesize that pancreas cancer cells produce BM proteins, such as type IV collagen, which then are secreted to the tumour surroundings and form basement membrane-like structures that the cancer cells can anchor to and by this stimulate cell growth, cell migration and perhaps avoid apoptosis.

Most importantly our study shows that a tumour stroma-derived substance could be a promising future tumour biomarker in evaluating prognosis and predict the effect of cancer treatment. This underlines the importance of expanding the horizon beyond the cancer cell, when searching for novel tumour biomarkers.

## Figures and Tables

**Table 1:** Patient characteristics

	**Control**	**Patients with pre- and postoperative samples**	**Patients with postoperative samples only**
Number	8	9^a^	5^a^
Sex (percentage of females/males)	62/38	56/44	60/40
Primary diagnosis (%)	Gallstone disease (75%)	Pancreas adenocarcinoma (78%)	Pancreas adenocarcinoma (100%)
	Diverticular disease (25%)	Papillary carcinoma (22%)	
Average age (years) (range)	51 (27–71)	64 (53–76)	71 (65–77)
*P*-value *vs* contol	—	0.06	—

a Postoperative samples from these groups together are included in the survival analysis (*n*=14).
